# Selective Activation of Retinal Ganglion Cell Subtypes Through Targeted Electrical Stimulation Parameters

**DOI:** 10.1109/TNSRE.2022.3149967

**Published:** 2022-02-17

**Authors:** Javad Paknahad, Mark Humayun, Gianluca Lazzi

**Affiliations:** Department of Electrical and Computer Engineering, University of Southern California, Los Angeles, CA 90089 USA; Departments of Ophthalmology and Biomedical Engineering, University of Southern California, Los Angeles, CA 90033 USA.; Departments of Ophthalmology, Electrical and Computer Engineering, and Biomedical Engineering, University of Southern California, Los Angeles, CA 90089 USA.

**Keywords:** Retinal prostheses, multi-scale computational modeling, high stimulation frequency, selective activation of RGCs

## Abstract

To restore vision to the low vision, epiretinal implants have been developed to electrically stimulate the healthy retinal ganglion cells (RGCs) in the degenerate retina. Given the diversity of retinal ganglion cells as well as the difference in their visual function, selective activation of RGCs subtypes can significantly improve the quality of the restored vision. Our recent results demonstrated that with the proper modulation of the current amplitude, small D1-bistratified cells with the contribution to blue/yellow color opponent pathway can be selectively activated at high frequency (200 Hz). The computational results correlated with the clinical findings revealing the blue sensation of 5/7 subjects with epiretinal implants at high frequency. Here we further explored the impacts of alterations in pulse duration and interphase gap on the response of RGCs at high frequency. We used the developed RGCs, A2-monostratified and D1-bistratified, and examined their response to a range of pulse durations (0.1−1.2 ms) and interphase gaps (0−1 ms). We found that the use of short pulse durations with no interphase gap at high frequency increases the differential response of RGCs, offering better opportunities for selective activation of D1 cells. The presence of the interphase gap has shown to reduce the overall differential response of RGCs. We also explored how the low density of calcium channels enhances the responsiveness of RGCs at high frequency.

## I. Introduction

Retinal implants have been developed to restore partial sight to patients who have been blinded by degenerative diseases such as retinitis pigmentosa (RP) and age-related macular degeneration (AMD). These devices convert visual information into spatiotemporal electrical stimuli patterns and aim at activating healthy retinal neurons [1]–[5]. In the early stages of degeneration, while photoreceptors are largely damaged, inter retinal neurons including retinal ganglion cells (RGCs) remain mostly intact [6]. Hence, retinal ganglion cells are often the primary target of electrical stimulation in epiretinal prosthetic systems.

Many attempts have been made towards improving the efficacy of current devices using both computational and experimental approaches. However, there are still many challenges that limit the spatial resolution of vision acquired through these devices. One of the most critical issues with epiretinal stimulation is the activation of RGCs axons of distant cell bodies [7]–[9]. Clinical studies of patients with epiretinal implants reported that a single stimulating electrode can result in perception of an elongated phosphene which is aligned with the RGCs axonal pathway [9].

Direct and indirect stimulations of RGCs have been proposed to avoid axonal activation and achieve a more focalized response from a population of RGCs [9]–[17]. Studies have shown that bipolar cells (BCs) can be preferentially activated using long pulse durations (25 ms) [9] or sinusoidal stimulation of low frequency (25 Hz) [11]. This indirect RGCs stimulation can avoid activating axons underneath electrodes and improve the spatial resolution of epiretinal implants. However, it has been reported that the response of RGCs to a train of stimulus pulses has been desensitized [12]–[15]. Research has been performed to directly stimulate RGCs using extremely short pulse durations in order to increase the stimulation threshold difference between the distal axon and the axon initial segment, and therefore reduce the chance for axonal excitation of RGCs [16]–[18].

Several studies have focused on the selective activation of RGCs to improve the quality of the restored vision. *Jepson et al*. [19] demonstrated the possibility of activating a single cell type without simultaneous activation of neighboring cells in the primate retina. High frequency electrical stimulation (≥ 1 kHz) with a proper current amplitude modulation has been shown to preferentially activate ON and OFF RGCs [20]–[23]. Further, the greater sensitivity of ON RGCs to electrical stimulation with long pulse durations relative to OFF RGCs has been observed, offering the stimulation strategy to selectively activate ON RGCs [24], [25]. While significant progress has been made towards preferential activation of ON and OFF RGCs, no stimulation strategies have been proposed to selectively activate morphologically different subtypes of RGCs [26], [27] and cells that carry specific types of visual information such as color and contrast [28]. For instance, small bistratified RGCs have been shown to contribute to blue/yellow color vision [29]–[31]. Also, clinical studies of subjects with epiretinal implants have demonstrated that blue color is perceived at high stimulus frequency [34]–[37]. The ability to characterize the response of RGCs to electrical stimulation and selectively target cells based on their morphological and physiological properties would represent a significant impact on the performance of current retinal prosthetic systems.

Recently, we developed morphologically and biophysically realistic models of the two classified RGCs, A2-monostratified and D1-bistratified [17], [32]. We validated these models by comparing them with the single-compartment models and experimentally recorded signals of the two cells using intracellular stimulations [33]. Using the typical stimulus parameters in Argus II devices, a symmetric charge-balanced with no interphase gap (IPG) and pulse width (PW) of 0.5 ms, we observed that D1 cells are more responsive to high frequency extracellular stimulation relative to A2 cells [32]. This differential response of RGCs offers potential for select activation of individual cells at high frequency (200 Hz). Our previous computational findings indicated that, with a careful selection of current amplitude, D1 small bistratified cells can be selectively targeted at high frequency with implications for encoding colors in future retinal prosthetic systems [34]. These results were verified with experimental data from the literature and correlated with the clinical data from the Argus II subjects [34]–[38].

In this work, using our combined Admittance method (AM)/NEURON multiscale computational platform [17], [34], [39]–[47], we further characterized the impacts of pulse duration and interphase gap on the differential response of RGCs at 200 Hz. To better understand the sensitivity of RGCs to different stimulus parameters, we analyzed the firing rates of cells as a function of modulations in current amplitude. Our results show that the difference in response between the two RGCs is highly dependent on modulations in both pulse durations and interphase gaps. Greater sensitivity of the A2 cell to the addition of interphase gap was observed compared to the D1 cell. We found that the addition of IPG reduces the likelihood for selective activation of RGCs at high frequency. Further, we investigated the role of calcium (Ca) channels on the responsiveness of RGCs at high frequency. The enhanced selectivity of RGCs can be achieved with a proper selection of the current amplitude at high frequency using short pulse durations with no IPG. This differential response of RGCs and therefore the ability to selectively target specific types of cells at high frequency can potentially help identify the mechanisms linked to different percepts and improve the effectiveness of current epiretinal prosthetic systems.

## II. Methods

**Fig. 1.**
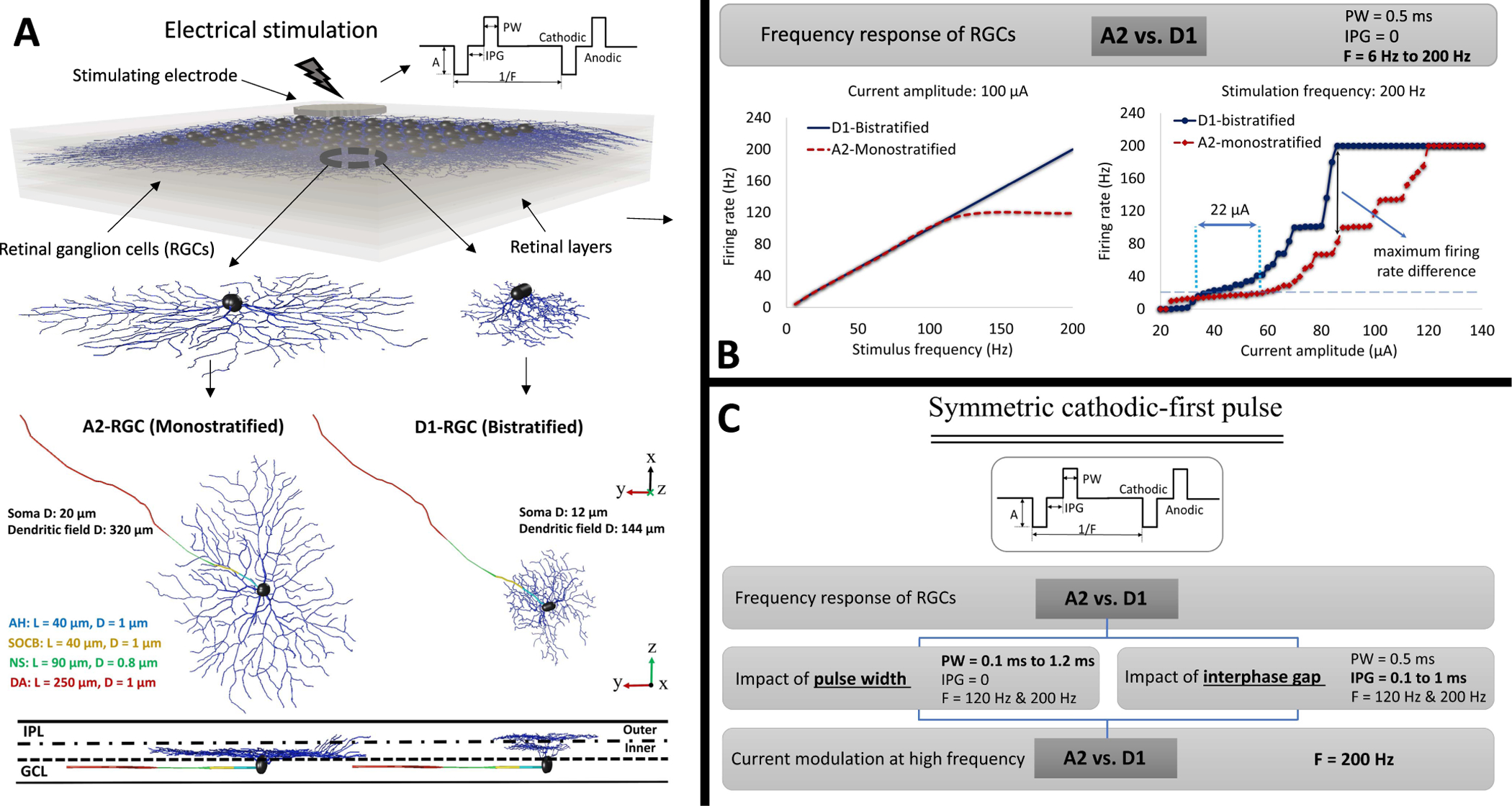
**A)** The constructed retina tissue model is populated with retinal ganglion cells 50 *μ*m beneath the stimulating electrode [17]. The A2 and D1 realistic morphologies are implemented and coded in our 3D multiscale Admittance Method/NEURON computational platform [39]–[47]. **B)** Frequency response of RGCs subtypes and the rate of response as a function of current amplitude at 200 Hz stimulation frequency. The computational results were verified with in-vitro experimental data and correlated well with clinical results with Argus II subjects [34], [38]. Further details are discussed at length in [17] and [34]. **C)** An overview of the analysis performed in this paper. Using a symmetric charge-balanced biphasic pulse, we first explored the impacts of modulations in pulse duration and interphase gap on the frequency response of RGCs. We compared the firing rate difference between A2 and D1 cells at high frequency over a range of pulse widths and interphase gaps. Then, the firing rates of RGCs over a range of current amplitude were compared and we characterized the effect of both PW and IPG on the response of RGCs at high stimulus frequency of 200 Hz. GCL: ganglion cell layer; IPL: inner plexiform layer; AH: axon hillock; SOCB: sodium channel band; NS: narrow segment; DA: distal axon; L: length of each band; D: diameter of each band.

A multi-scale computational model has been utilized that allows constructing a voxel model of the bulk retina tissue, and the components of implantable devices. This computational platform was adapted to predict the electric fields generated inside the retina tissue, coupled to a multi-compartmental model of neurons in order to determine the activation of biophysically and morphologically realistic RGCs through targeted electrical stimulation [39]–[47]. The two developed RGCs, A2-monostratified and D1-bistratified, and the level of their dendritic ramification are shown in Fig. 1a. The morphology of RGCs was extracted as SWC files from the NeuroMorpho dataset [48], [49] and imported to NEURON [50]. The A2 cell has soma and dendritic field diameters of 20 *μ*m and 320 *μ*m, respectively and the D1 cell has soma and dendritic field diameters of 12 *μ*m and 144 *μ*m, respectively [51]. The parameters of RGCs axon are adapted from [52]. A stimulating electrode of 200 *μ*m diameter is placed on the top-center of the retinal ganglion cell layer in the bulk retina tissue and is positioned 50 *μ*m from the cell body of the RGCs. The ionic channel conductance values of both A2 and D1 cells for the soma, dendrites, and axon are listed in Tables I and II. More details on the NEURON modeling of these RGCs and AM simulations are discussed in [17], [32], [34]. The impacts of morphological variations including the sodium channel band (SOCB) length, soma diameter, and axon diameter as well as the position of the electrode with respect to cells and along the axon on the differential response of the two subtypes of RGCs have been comprehensively discussed in our previous works [17], [34]. Given the greatest excitability of the D1 cells to high stimulation frequency (Fig. 1b), we only centered our focus on the role of PW and IPG in the response of RGCs to electrical stimulation, particularly at high frequency.

The primary goal of this study is to determine the effects of different electrical stimulation parameters on the frequency response of RGCs. We used symmetric charge-balanced biphasic waveforms in all performed simulations to avoid charge accumulation and damage to the tissue. We previously used the typical stimulus waveform in Argus II devices (symmetric cathodic-first with no interphase gap and 0.5 ms pulse duration) [53]. Here we evaluated the impacts of a range of pulse durations from 0.1 ms to 1.2 ms, and interphase gaps ranging from 0 to 1 ms on RGCs frequency response. To assess the possibility of enhancing selective activation of RGCs, the response of the cells over a range of current amplitudes was compared at 200 Hz as shown in Fig. 1c.

The influence of pulse width and interphase gap on RGCs response to electrical stimulation has proven in the literature [54], [55]. Therefore, a fixed current amplitude cannot be used for comparing the RGCs frequency response of varying pulse durations and interphase gaps. It is important to eliminate the effects of stimulus threshold (charge threshold) difference among different pulse widths and interphase gaps on RGCs firing rate. A proper current amplitude needs to be set for a given electrical stimulation parameter before any comparisons between the responsiveness of RGCs to high stimulation frequency. Therefore, we defined the current amplitude as the minimum current required to achieve the spike probability (the total number of spikes divided by the total number of delivered stimulus pulses) of 100% at a lower frequency of 120 Hz, the frequency tested in clinics [38], in both A2 and D1 cells for a range of pulse durations and interphase gaps. Then, the adjusted current associated with each pulse duration and interphase gap was used to illustrate how the two RGCs can maintain their response at 200 Hz stimulus frequency. This strategy could minimize the number of plots and analysis required to evaluate the impacts of several pulse durations and interphase gaps on the response of RGCs over a range of frequencies.

We further compared the firing rate difference between the two cells as a function of pulse amplitude for the two pulse durations of 0.1 ms and 1 ms without the IPG. Similarly, we analyzed the differential response of RGCs for a pulse duration of 0.5 ms without and with the IPG of 1 ms as a function of current amplitude. Fig. 1c provides an overview of the analysis performed in this paper. All the simulations were done using the AM-NEURON computational platform [39]–[47]. Further details of this modeling framework are described in [17], [34], [39]–[47].

## III. Results

### A. Frequency Response: Pulse Width Modulations

**Fig. 2.**
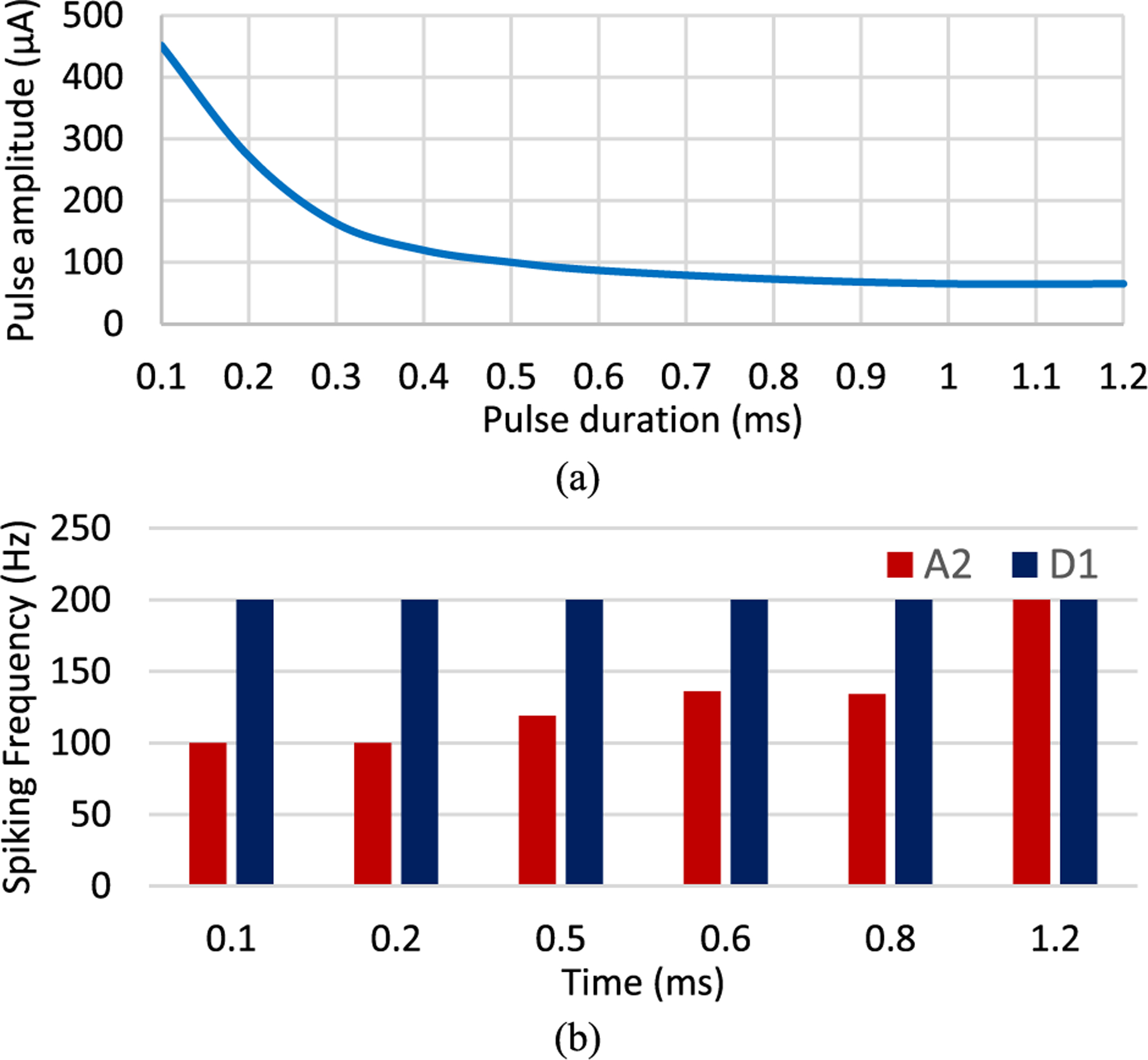
(a) The minimum current amplitude required to achieve 100% spike probability at 120 Hz (120 Hz firing rates) for a range of pulse durations and no IPG, (b) the difference in frequency response between the A2 and D1 RGCs at 200 Hz. Results demonstrate that the firing rates of cells can be better differentiated using shorter pulse widths.

We analyzed the influence of pulse width variations from 0.1 to 1.2 ms on the frequency response and the level of excitability of the two RGCs at high frequency. Fig. 2a shows the tuned current amplitude for each pulse width to achieve 100% spike probability at 120 Hz in both cells. As expected, the strength-duration curve is decaying exponentially, and the curve flattens out at around 0.8 ms pulse duration. The current associated with each pulse duration was used to compare the responsiveness of RGCs to 200 Hz stimulus frequency. Fig. 2b shows the spiking frequency of D1 and A2 cells as a function of pulse duration. Responses of both cell types illustrate that pulses with shorter durations result in greater differentiation in RGCs firing rate at high frequency, although the required current is higher. This differential response became smaller and both RGCs almost follow a similar rate of firing as the pulse duration is increased.

### B. Frequency Response: Interphase Gap Modulations

**Fig. 3.**
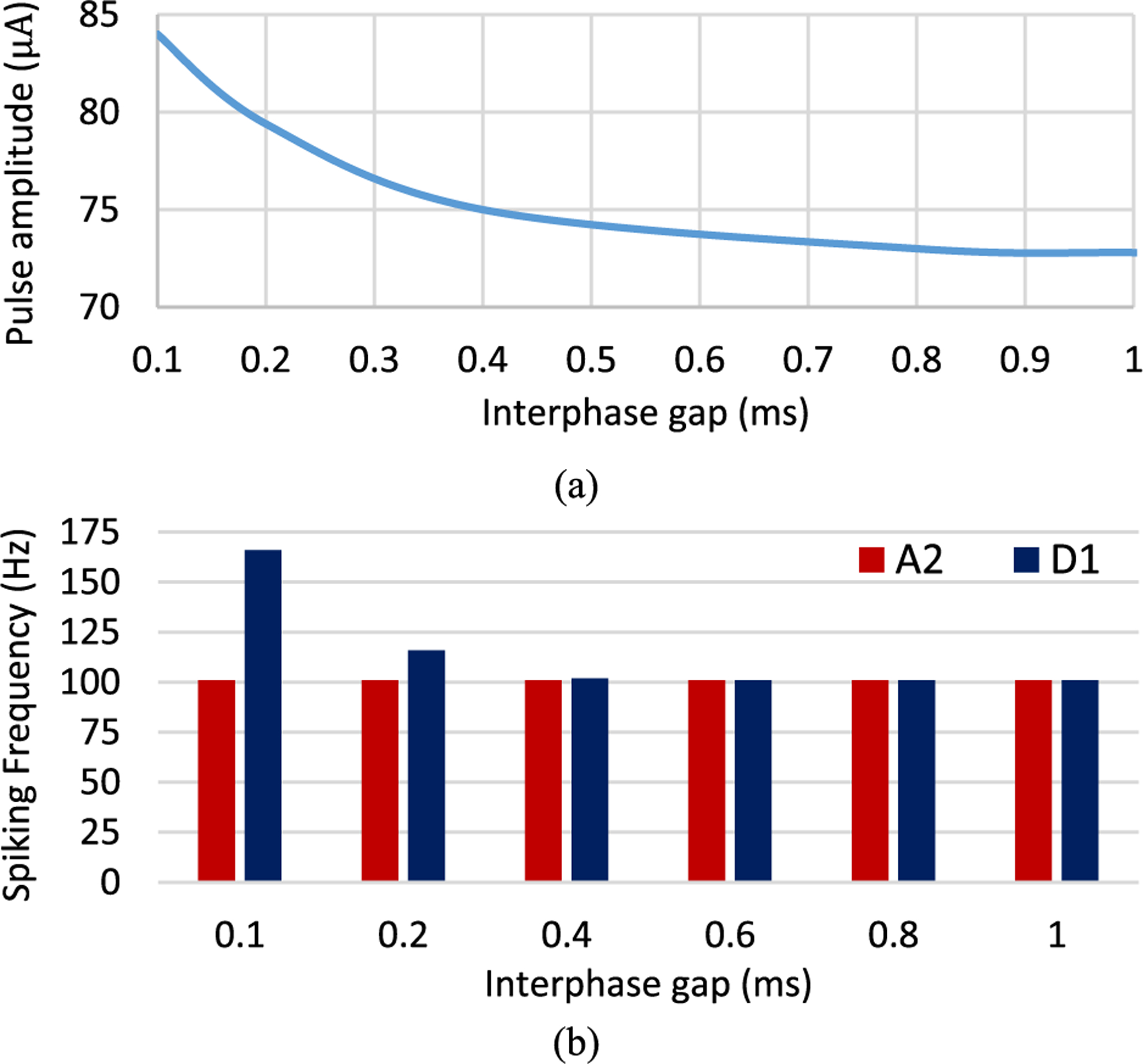
(a) The minimum current amplitude required to achieve 100% spike probability at 120 Hz (120 Hz firing rates) for a range of IPGs and PW of 0.5 ms; (b) The difference in firing rate between A2 and D1 RGCs at 200 Hz for a range of interphase gaps. Results show that even though the addition of interphase gap decreases the stimulation threshold of RGCs, it does not increase the differential response of cells.

So far, we assumed that there is no interphase gap in the stimulation waveform. Here we examined the change in the differential response of RGCs in the presence of IPGs at high frequency (200 Hz). We compared the responsiveness of A2 and D1 cells to modulations in IPGs. Fig. 3a illustrates a decrease in the current amplitude with increasing the interphase gap up to around 0.8 ms. However, the effect of IPG on the current amplitude is negligible at values higher than 0.8 ms. A comparison of firing rate difference between the D1-bistratified and A2-monostratified RGCs with variations in the interphase gap ranging from 0.1 to 1 ms is represented in Fig. 3b. Interestingly, the difference between the excitability of these cells at high frequency was reduced and converged to zero at IPGs greater than 0.2 ms. Our observation of the similarity of the two RGCs responsiveness to longer IPGs suggests that the likelihood for preferential activation of cells is reduced in the presence of IPG.

### C. Current Modulations: Effects of Pulse Width on RGCs Response

**Fig. 4.**
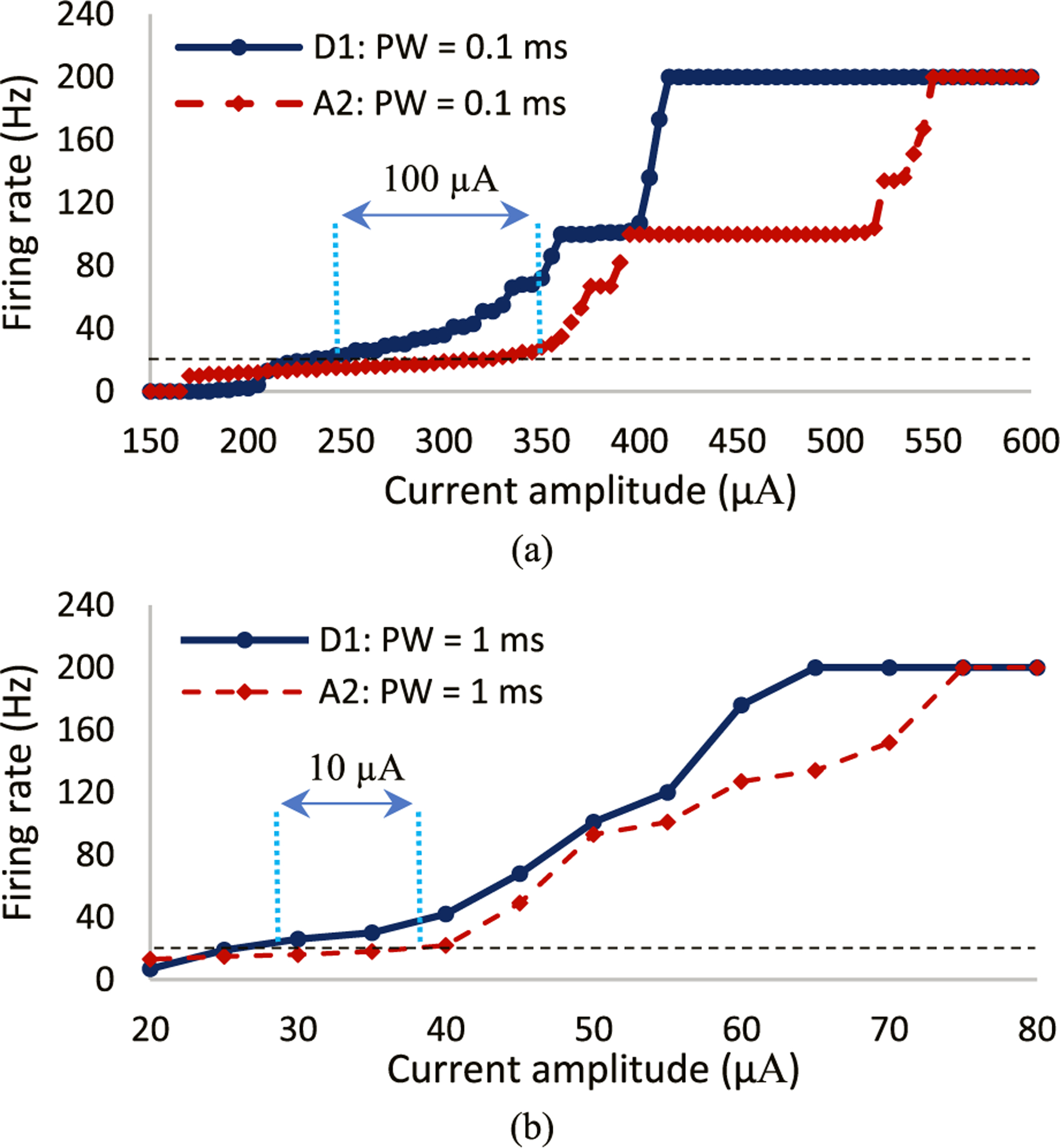
Firing rate as a function of pulse amplitude for both A2 and D1 cells at 200 Hz stimulation frequency with no IPG: (a) short pulse duration of 0.1 ms; (b) long pulse duration of 1 ms. Data represent the impacts of pulse width on the response of A2 and D1 RGCs. Results demonstrate that the differential responsiveness of RGCs is higher using shorter pulse durations. This suggests the greatest stimulus threshold difference and a better chance of selective activation of D1 cells using short pulse width with a proper selection of current amplitude at high frequency.

To better understand the sensitivity of RGCs to high stimulation frequency of varying pulse durations, we plotted the cells firing rate as a function of current amplitude at 200 Hz. The current is incremented every 5 *μ*A and the number of spikes per second are recorded for both cells. The A2 and D1 response curves for the impulse of 0.1 ms width and current amplitudes ranging from 150 to 600 *μ*A are shown in Fig. 4a. Overall, the greater difference in the rate of response between the cells can be seen using short pulse durations. As we increase the pulse amplitude, D1 cells show a higher probability of excitation at higher firing rate relative to A2 cells.

Our computational modeling further reveals that there is a wide saturation window at 100 Hz firing rate (50% spike probability) for both cells. This window is almost three times larger for the A2-monostratified cell relative to the D1-bistratified cell (Fig. 4a). This also leads to the great suprathreshold difference between the cells to achieve 200 Hz spiking rate (100% spike probability), indicating that A2 cells are less responsive at high frequency. Fig. 4b compares the firing rate of both RGCs as alterations in pulse amplitude ranging from 20 to 80 *μ*A for the longer pulse duration of 1 ms. While a slower rate of response in A2 cells can be seen here as well, the differential response of cells is significantly lower compared to short-duration pulses. No saturation window was observed with long stimulus pulse durations. Although the level of current amplitude is lower using long pulse durations, the charge threshold appears to be greater compared to pulses with short durations (See Fig. 4a and b). For example, the minimum current to achieve 200 Hz firing rate in A2 cells for the short pulse width is 550 *μ*A, indicating 55 nC charge. However, the current and charge for the long pulse duration (1 ms) are 75 *μ*A and 75 nC, respectively. This offers a plausible explanation that the close sensitivity of the two RGCs to long stimulus pulses can be due to the large charge threshold associated with long pulse durations.

The typical stimulation frequency applied in epiretinal prosthetic systems and particularly Argus II is 20 Hz (to achieve stable phosphene perception) [53]. Therefore, with a proper modulation of current amplitude at 200 Hz stimulus frequency, the spiking rate of RGCs can be controlled to remain at 20 Hz (similarly to the current range of RGCs’ firing rate). This technique leads to the elevated differential response of RGCs and the enhanced possibility for preferential activation of RGCs. Fig. 4 indicates that the current amplitude difference between the two cells to reach 20 Hz firing rate is much larger using the short 0.1 ms pulse duration (~100 *μ*A) relative to the long 1 ms pulse duration (~10 *μ*A), offering a greater chance for selective activation of D1 cells using short pulse durations.

### D. Current Modulations: Effects of Interphase Gap on RGCs Response

**Fig. 5.**
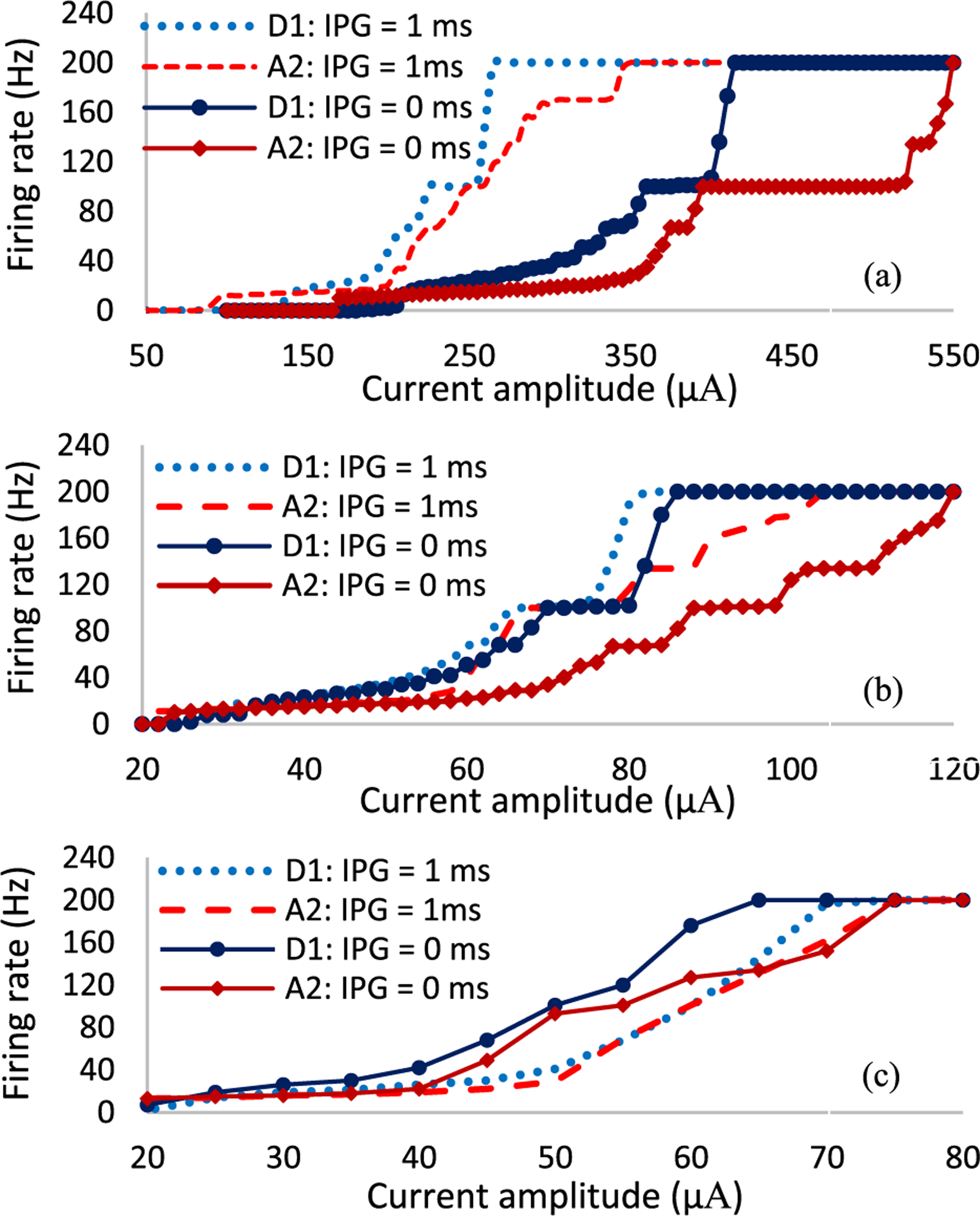
Firing rates of A2 and D1 cells as a function of pulse amplitude at 200 Hz stimulus frequency for both the presence (dash lines) and absence of IPG (solid lines). (a) PW = 0.1 ms; (b) PW = 0.5 ms; (c) PW = 1 ms. Results show that the differential response of RGCs is reduced with the inclusion of an interphase gap of 1 ms, lowering chance of RGCs select excitation with the inclusion of IPGs.

To better examine the effect of interphase gap on RGCs response, we plotted the firing rate of both A2 and D1 cells as a function of current amplitude for three pulse widths of 0.1, 0.5, and 1 ms with and without the presence of IPG (IPG = 1 ms) at 200 Hz as shown in Fig. 5. The addition of IPG and pulse width alterations significantly modulate the response of RGCs with increasing current amplitude. The short pulse width of 0.1 ms with IPG of 1 ms reduces the stimulation threshold and enhances the excitability of RGCs at high frequency (Fig. 5a). However, the presence of IPG decreases the differential response of cells and the likelihood of selective activation of RGCs for all pulse widths. The reduced threshold is less pronounced using the PW of 0.5 ms (Fig. 5b). Fig. 5c shows that the responsiveness of RGCs at high frequency is reduced using long PW and IPG of 1 ms. This agrees with results from the literature, showing the blocking effect of high stimulation frequency with long IPGs [55]. The anodic phase of the stimulus waveforms with long PWs and IPGs hyperpolarizes the membrane, therefore increasing the current amplitude required for eliciting an action potential for the following cathodic stimulation phase. Comparing the solid and dashed lines in Fig. 5 illustrates that the addition of IPGs decreases the differential response of RGCs, reducing the likelihood for selective activation of RGCs at high frequency.

**Fig. 6.**
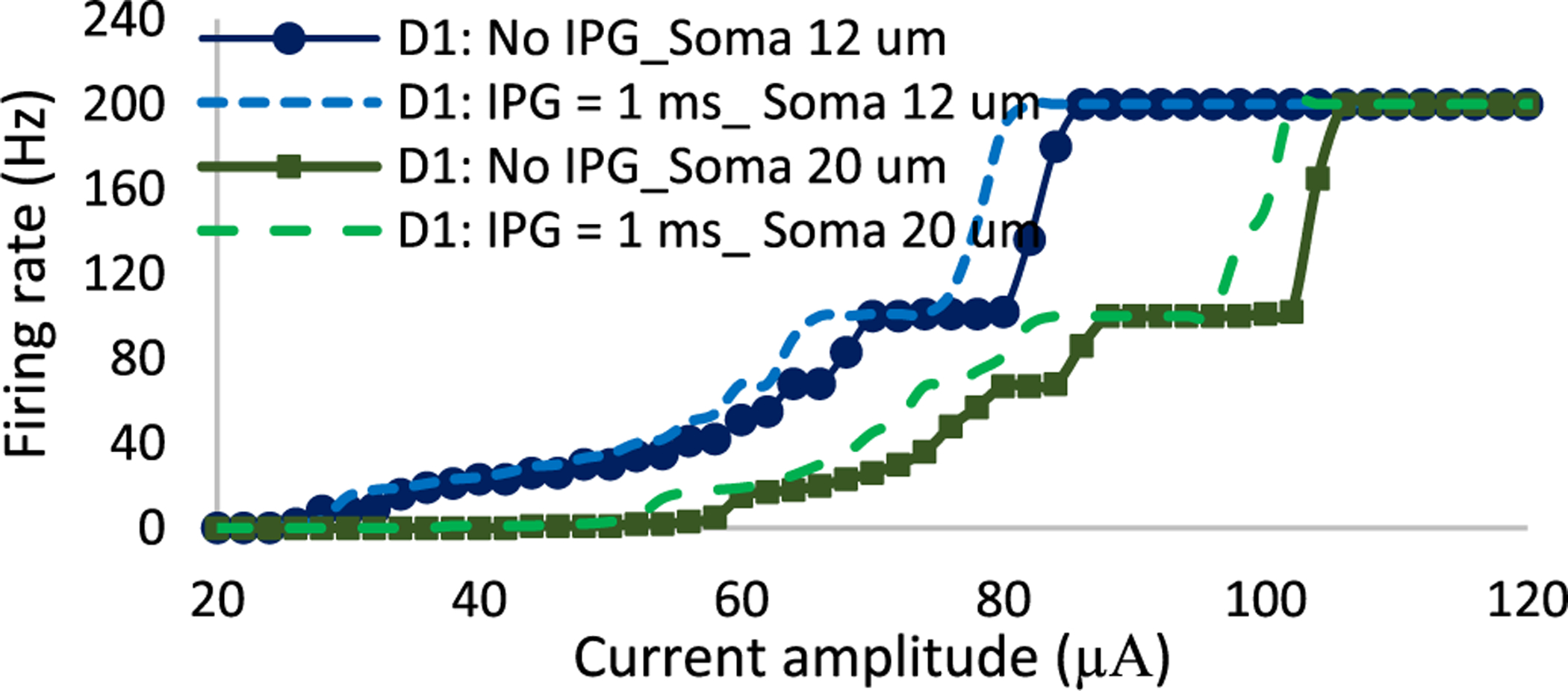
The impact of soma diameter on RGCs sensitivity to the addition of IPG = 1ms and PW of 0.5 ms at 200 Hz. Response curves of small and large D1 cells with and without the presence of IPGs are shown by dash and solid lines, respectively. Data show that soma size changes do not significantly influence RGCs responsiveness to alterations in IPG.

A2 cells have a larger soma diameter compared to D1 cells [26]. Therefore, we tested whether the soma diameter difference between the two cells plays a role in the decreased differential response of RGCs. We altered the soma diameter of the D1 cell to 20 *μ*m and compared the results with the original 12 *μ*m soma size of this cell. Fig. 6 compares the firing rates of small and large D1 cells with modulations in current amplitude in the presence (IPG = 1 ms) and absence of IPGs. Results show that cells with large soma sizes are less responsive to high stimulation frequency as the response curve is shifted to the right, indicating the higher activation threshold of large cells. The figure further indicates that soma size has a negligible effect on RGCs response sensitivity to the addition of IPGs.

### E. Impacts of Biophysics on RGCs Response

**Fig. 7.**
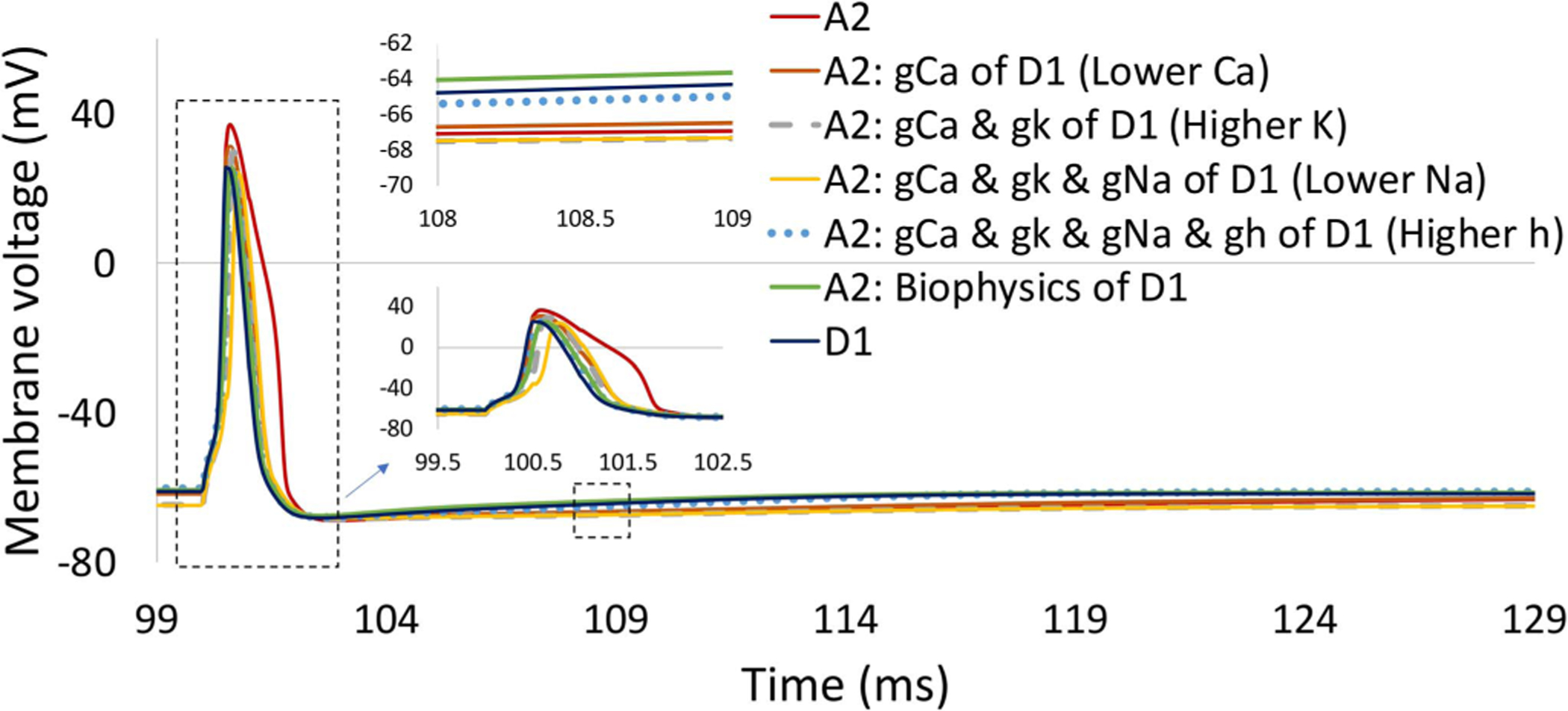
The influence of maximum ionic membrane conductance difference between the two cells on the elicited action potential. Results indicate the significant impact of calcium (Ca) channel density on RGCs spike width, and the influence of hyperpolarization-activated (h) channel on RGCs refractory period.

We further delved into other potential underlying mechanisms contributing to the differential sensitivity of RGCs to high stimulation frequency in the presence and absence of IPGs. Fig. 7 demonstrates the effect of the biophysical properties of the two RGCs on the elicited action potential. We first changed the maximum ionic conductance of each channel in the A2 cell to that of the D1 cell based on the ionic properties provided in Table I. This allowed us to examine the role of each channel in shaping the response of the two cells to electrical stimulation. We found that reducing the density of Ca decreases the spike width (Fig. 7). An increase in the potassium (K) density modulates the resting membrane potential to a lower value. Sodium (Na) does not play a major role in shaping the response of the cell and reducing the Na conductance slightly enhances the response latency of the cell. The addition of the hyperpolarization-activated channel (h) to the A2 cell, changing the ionic conductance value from 0 to 0.0001 S/cm2 (Table I), reduces the refractory period of the A2 cell approximately from 25 ms to 18 ms (red vs. blue round dot curves). This agrees with the experimental data observing a longer refractory period with the lower value of Ih current [56]. Taken together, Ca and h channels play significant roles in the firing pattern difference between the A2 and D1 cells. Intrinsic electrophysiological differences among RGCs and between these two classified cells have been shown in the literature [33]. For instance, the depolarizing sag, the change in the membrane potential from the onset to the end of an intracellular hyperpolarizing step current, has been shown to be greater for D1 cells compared to A2 cells [33]. Further, the low density of h channels has been shown to reduce the depolarizing sag and enhance the refractory period of pyramidal neurons [56].

### F. Role of Ca Channels in RGCs Excitability at High Frequency

**Fig. 8.**
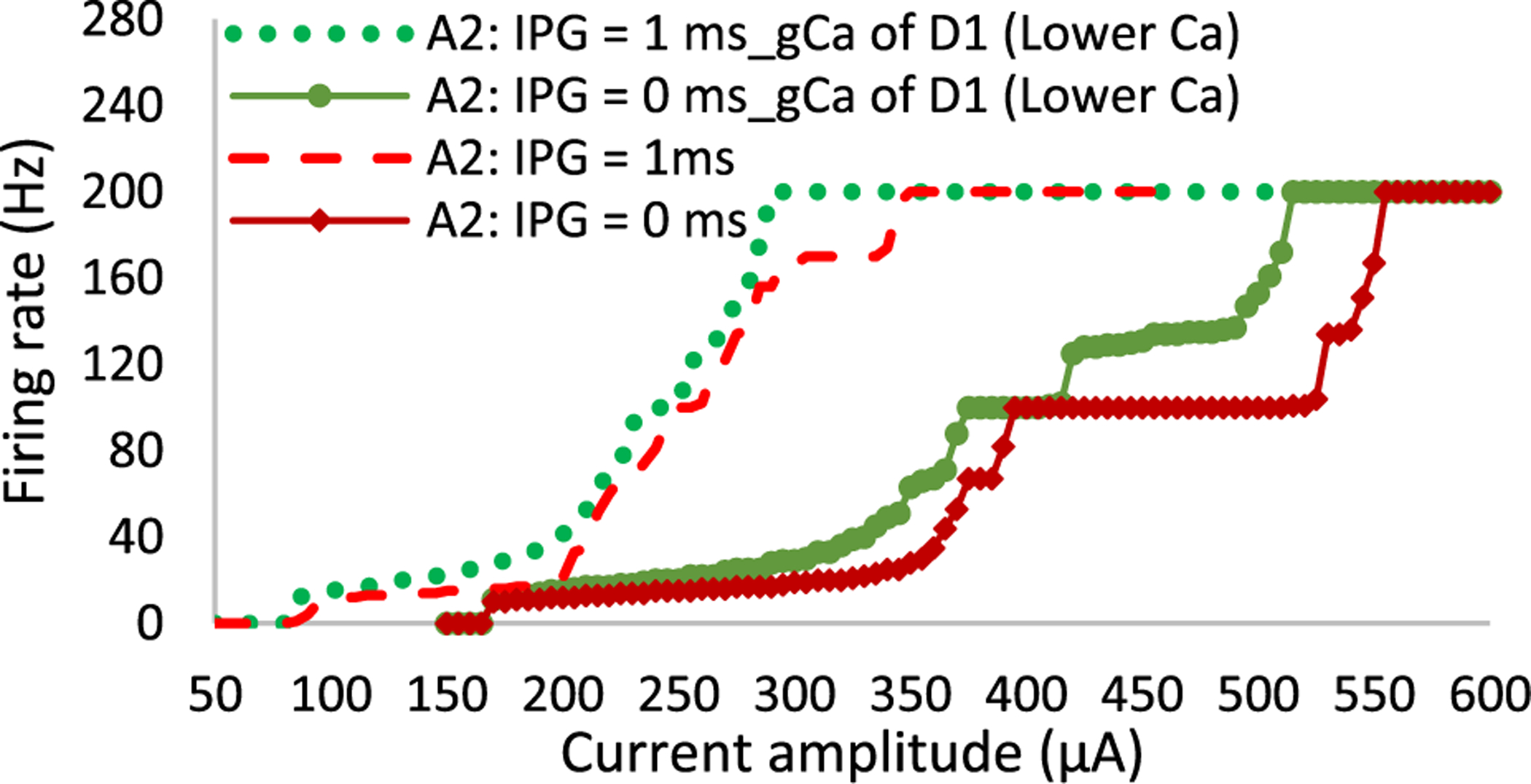
The role of Ca channel in excitability of RGCs at high frequency for a pulse duration of 0.1 ms. Data show that reducing the density of Ca channel of the A2 cell to the Ca conductance value of the D1 cell enhances the excitability of the cell and decreases the saturation window in the absence of IPG. However, in the presence of IPG the influence of the Ca channel on the cell response is less pronounced.

Given the significant contribution of Ca channel density to the differential firing pattern of RGCs, we examined the role of this channel in the greater saturation window and lower responsiveness of the A2 cell compared to the D1 cell at high frequency (Fig. 4). We also investigated the reduced differential response of RGCs due to the addition of IPG (Fig. 5). Fig. 8 compares the firing rate of A2 cells as a function of current amplitude for the pulse duration of 0.1 ms at 200 Hz in the presence and absence of IPG = 1 ms. We investigated alterations in the cell response as the maximum Ca conductance of the A2 cell was reduced to the value of the D1 cell. Lowering the Ca density channel augments the responsiveness of the cell and further reduces the saturation window of the cell. Fig. 8 further illustrates that only by modulating the Ca channel density the reduced differential response of RGCs in the presence of IPG can be predicted. Therefore, our computational findings indicate the role of the difference in the Ca density of RGCs in the excitability of cells and lowered differential response of RGCs with the addition of IPG.

## IV. Discussion

Using our combined AM-NEURON multi-scale computational platform [39]–[47], we developed morphologically and biophysically realistic models of two classified RGCs, A2- monostratified and D1-bistratified [32]. This modeling framework has enabled us to have a more precise prediction of retinal neuron activities due to electrical stimulation [34], and particularly in this work the response of RGCs to epiretinal electrical stimulation of various stimulation parameters. We assessed the impact of pulse duration and interphase gap in a symmetric cathodic-first biphasic waveform on the frequency response of RGCs, and their responsiveness to high stimulus frequency. We have explored different aspects of RGCs response sensitivity to varying stimulus pulse widths and interphase gaps. Our computational findings reveal that modulations in pulse durations and involvement of interphase gap can significantly influence the differential firing rates of RGCs and the possibility of selectively activating RGCs at high stimulation frequency.

### A. Impacts of PW and IPG on Frequency Response of RGCs

Several studies have focused on the responsiveness of RGCs to high stimulation frequency. However, conflicting results have been reported regarding the ability of RGCs to maintain their response at high rates of electrical stimulation. While it has been shown that RGCs can manage to spike at a high stimulation frequency of 250 Hz with direct stimulation [57], [58], *Jensen et al*. observed that RGCs cannot maintain their firing rates at stimulus frequencies higher than 40 Hz [59]. *Cai et al*. [60] further demonstrated that the responsiveness of RGCs to a range of stimulation frequencies from 100 to 700 Hz varies across cell types. Even within one morphological subtype of RGCs, small cells have shown to be more responsive to high frequency relative to large cells [54]. These findings suggest that different types of RGCs may experience differences in their response at a high stimulation frequency of varying electrical stimulation parameters.

In the present study, we investigated the effects of pulse duration and interphase gap on the frequency response of A2 and D1 RGCs. To this aim, we adjusted the current amplitude of each pulse width and interphase gap to achieve one spike per stimulus pulse (100% spike probability) at 120 Hz. Using the tuned current amplitude, the firing rates of cells at 200 Hz for a range of pulse widths and interphase gaps were computed. One may ask why we did not adjust the current amplitude to obtain an identical level of charges for each delivered stimulus pulse. The reason behind using this strategy is the fact that charge threshold varies across pulse durations and a shorter pulse duration leads to a lower charge threshold [17], [61]. Therefore, the equal charge assumption leads to higher rates of spikes at extremely shorter pulse widths relative to long pulse durations [62] and makes it difficult to compare the differential response of RGCs over a range of pulse durations at high stimulus frequency. We found this approach to be more effective in eliminating the effects of stimulus threshold difference among various pulse durations and interphase gaps on the responsiveness of RGCs.

Consistently with previous reports [54], [55], the adjusted current amplitudes exponentially decay with an increase in both pulse duration and interphase gap. The strength-duration and -interphase gap curves flatten out at approximately 0.8 ms and 0.6 ms, respectively as shown in Fig. 2a and 3a. Our results further show that shorter pulse durations can increase the differential response of RGCs. While the addition of an interphase gap can reduce the stimulus threshold, the IPG appears to minimize the firing rate difference between the two cells at high frequency.

### B. Selective Activation of D1 Cells with Short Pulse Width

ON and OFF RGCs have been known to be the key components of signaling in the normal retina for relaying visual information to the brain. In natural signaling of the retina, ON and OFF RGCs do not fire simultaneously due to their exclusive response to light increments and decrements [63]. Therefore, several stimulation strategies have been reported to selectively activate ON and OFF RGCs. High stimulation frequency (≥ 1 kHz) of varying current amplitudes has been used to preferentially target ON and OFF RGCs [22]–[23]. Indirect stimulation has shown to result in a closer correlation between the electrically-elicited and light-elicited response of ON RGCs relative to OFF RGCs, suggesting more physiologically realistic signals by targeting ON RGCs [64]. Accordingly, there have been more recent attempts towards selective activation of ON RGCs and increasing the ON/OFF RGCs response ratios by modulating stimulation frequency, pulse duration, and stimulus charge [24], [25].

Despite these efforts, selective activation of a broader range of morphologically different RGCs has remained a significant challenge. Retinal ganglion cells are not only limited to ON, OFF, and ON-OFF types and there is a wide range of morphologically different subtypes of RGCs [26], [27] that convey specific types of visual information to the brain [28]. *Jepson et al*. [19] found that spatial selectivity of RGCs can be achieved using a small stimulating electrode (10 *μ*m) in the primate retina. However, this single RGC activation without simultaneous activation of nearby cells depends on the position of the stimulation electrode. Large charge density associated with the small diameter of the electrode can damage the retina tissue as well [65]. The ability to selectively target individual RGCs subtypes with the current stimulation electrode which covers a large number of RGCs would significantly improve the performance of epiretinal implants.

Therefore, we characterized how a change in pulse duration and interphase gap in a symmetric cathodic-first biphasic waveform can influence the potential for more selective excitation of individual RGCs at high frequency. Assuming RGCs firing rate of current epiretinal implants to be approximately 20 Hz (typical stimulation frequency of Argus II [53]), the greatest current amplitude difference to reach 20 Hz firing rate in both cells can be achieved using a short pulse duration of 0.1 ms at 200 Hz (Fig. 4a). This leads to the greater likelihood of preferential activation of D1-bistratified RGCs over A2-monostratified RGCs. On the contrary, the firing rate difference between the two cells and therefore the likelihood for selective activation were reduced using the long pulse duration (1 ms) as shown in Fig. 4b.

### C. Mechanisms Underlying RGCs Response at High Stimulation Frequency

The underlying mechanisms affecting the significant difference in RGCs differential response to pulse duration modulations can be related to the difference in the total delivered charge between long and short duration pulses. We previously observed a certain spike latency in the time course response of the A2 cell at 200 Hz, which this latency was not significant in D1 cells [34]. This latency in spike has shown to be another factor in addition to the soma diameter contributing to the less excitability of A2 cells and therefore slower rate of firing rate changes with an increase in current amplitude. This is in line with the experimental data showing RGCs with long spike latencies are less responsive to high stimulus frequencies [58]. As a result, a reduction in spike latency can improve the ability of RGCs to maintain their response at high frequency. Interestingly, it has been shown that a high level of delivered charge leads to lower averaged spike latency of RGCs [62]. Together, on average, greater stimulus charge thresholds associated with long pulses compared to short pulses play an essential role in the reduced differential response of RGCs and consequently the lower chance for selective activation of RGCs.

**Fig. 9.**
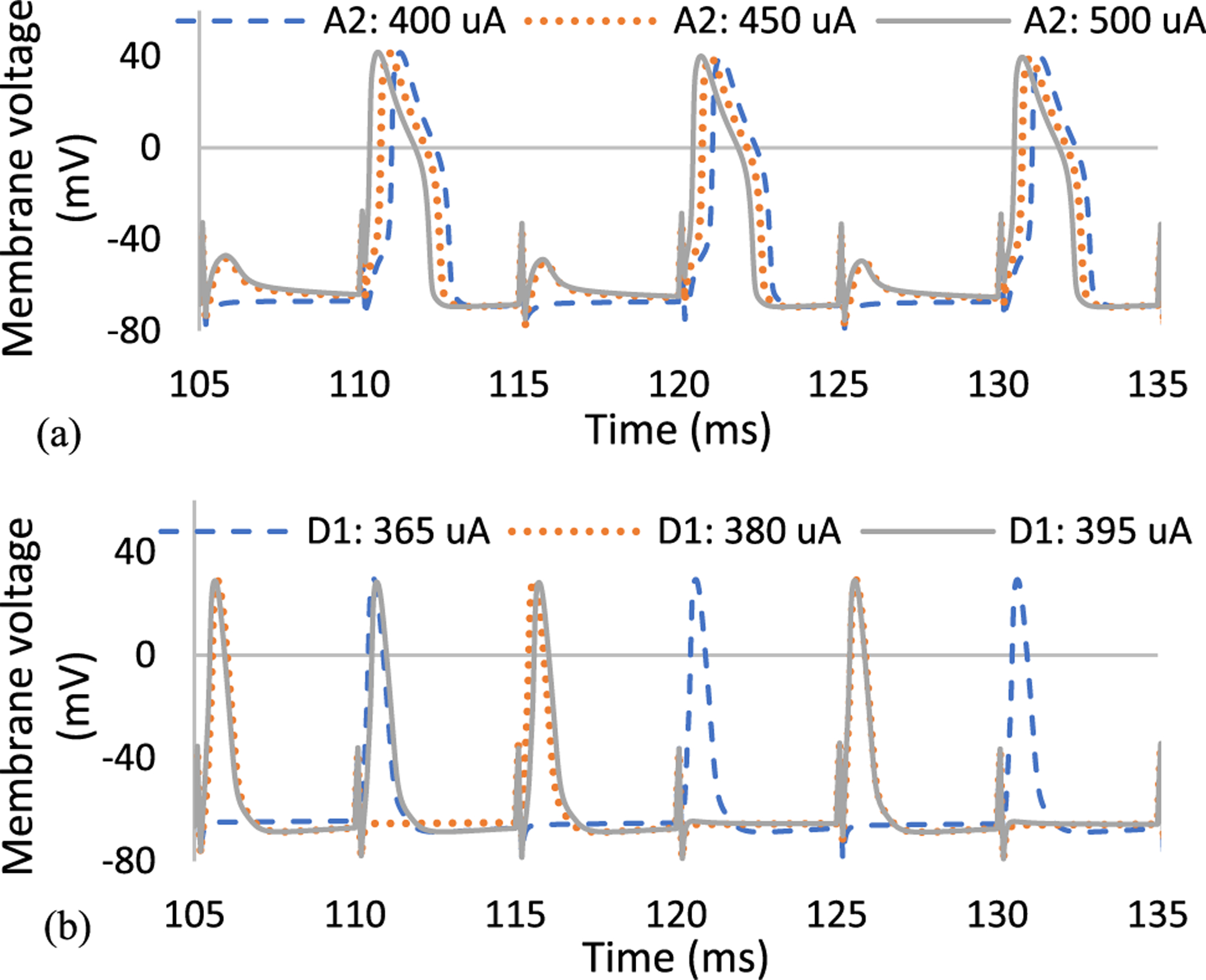
Membrane voltage recorded from the cell body in response to electrical stimulus pulses of 0.1 ms in duration during the saturation window of (a) A2 cell; (b) D1 cell as shown in Fig. 4a. The small spikes were observed in the A2 cell response with lower responsiveness at a high frequency relative to the D1 cell.

A long saturation window was observed in the A2 cell compared to the D1 cell using the short pulse width of 0.1 ms and no IPG (Fig. 4a). This saturation effect is in agreement with experimental data on RGCs responsiveness at high stimulus frequencies, indicating that some RGC subtypes cannot follow high rate of stimulus pulses even with increasing current amplitude [60]. We found that the lower Ca density of the D1 cell relative to the A2 cell contributes to this period of unchanged firing rates of the A2 cell with an increase in the current amplitude (Fig. 8). Fig. 9 compares the membrane voltage of the two cells within their current window of saturation in response to a short stimulus pulse of 0.1 ms according to Fig. 4a. While increasing the current amplitude does not change the number of elicited spikes in the A2 cell, it generates small spikes following every standard action potential (Fig. 9a). The small waveforms are not elicited in the D1 cell with the greater responsiveness at high frequency as shown in Fig. 9b. Our computational results correlate well with the electrophysiological experiments measuring the response of different RGCs subtypes to high stimulation frequencies up to 700 Hz [60]. The small waveforms were recorded in all types of RGCs except the brick transient (BT) cell with the highest ability to follow high rates of stimulus pulses [60].

Fig. 9a further supports the role of injected charge in the response latency of the cell. An increase in the current amplitude and therefore the injected charge reduces the spike latency of the A2 cell. We also found that the difference in the density of the Ca channel can impact the differential sensitivity of RGCs to high stimulation frequency (Fig. 8). Further, the longer refractory period of the A2 cell compared to the D1 cell due to the lower sag and absence of h channel (Table I) may influence the ability of the cell to follow high rates of stimulus pulses. We could not examine the role of hyperpolarization-activated (h) ionic channel in RGCs responsiveness in isolation because of modulations in the resting membrane potential and activation threshold of RGCs with changes in the h maximum conductance value.

### D. Sensitivity of RGCs to The Presence of IPG

Our computational modeling indicates that the presence of an interphase gap decreases the averaged differential response of A2 and D1 cells. Even though the addition of IPG reduces the stimulation threshold, the overall potential of Selective activation of RGCs is declined. We also observed that the inclusion of IPG leads to greater changes in the response of A2 cells relative to D1 cells. Similarly to the role of stimulus charge on spike latency, we hypothesize that IPGs can also influence the spike timing of RGCs per each stimulus pulse. It is well-known that the addition of IPG reduces the activation threshold of not only RGCs [54], [55], but also auditory nerves [66]. This arises from the fact that voltage-gated sodium channels require more time for depolarization events before reversing the polarity of stimulation, thereby affecting the stimulus threshold, and possibly leading to the delay in eliciting action potentials. Therefore, the addition of IPGs decreases the spike latency observed in A2 cells, leading to a greater rate of changes in firing rate with an increase in current amplitude relative to that of D1 cells (Fig. 5).

**Fig. 10.**
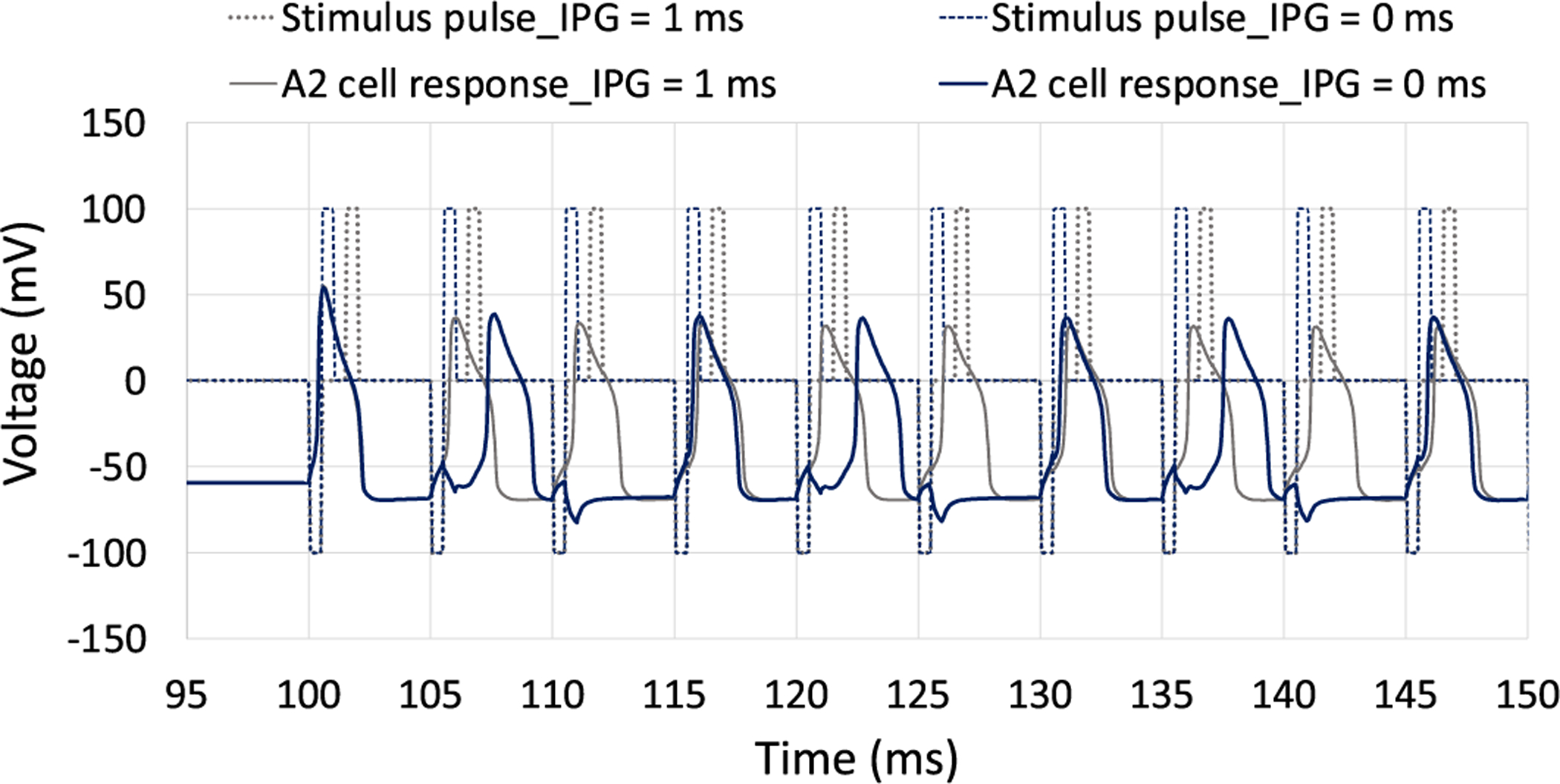
Comparison of the membrane voltages resulting from symmetric cathodic-first biphasic waveforms with and without the presence of interphase gap. The current amplitude is set to 100 *μ*A and PW is 0.5 ms. Data show that the A2 cell can maintain one spike per each stimulus pulse with the inclusion of IPG, while this is not the case when the IPG is not present.

Fig. 10 compares the membrane potential of the A2 cell for symmetric cathodic-first biphasic pulses with and without the inclusion of IPG at 200 Hz. The current amplitude is set to 100 *μ*A and pulse width is 0.5 ms. Results indicate that the IPG helps the A2 cell elicit spike per each stimulus pulse, thereby enhancing the firing rate of this cell type. We showed that both small and large cells response have a similar sensitivity to the addition of IPG and no obvious correlations with soma diameter changes were found (see Fig. 6).

### E. Implications for Clinical Applications

Various electrical stimulation strategies have been proposed to improve the effectiveness of current epiretinal prosthetic systems. However, there are challenges associated with many systems: i) they are not currently implementable clinically; ii) more likely can damage the tissue using asymmetric waveforms, symmetric long pulse durations, and small electrode sizes. In this work, we used the same stimulating electrode in current epiretinal implants and limited our study to the clinically applicable range of electrical stimulation parameters. We proposed electrical stimulation strategies that not only enhance focalized activation of RGCs [16]–[17], but also effectively increase the chance for selective activation of RGCs subtypes.

The differential response of RGCs discussed in this work can be linked to different percepts such as color and contrast. Recent clinical testing has revealed that Argus II subjects perceived some variations in color perceptions particularly at high stimulation frequency [34], [38]. Our most recent computational results were well correlated with this clinical data and verified with the in-vitro experiments on RGCs [34], [54]. The simulation results of this paper further show the potential of maximizing the differential response of RGCs using the currently developed epiretinal prosthetic system. Modeling two subtypes of RGCs allowed us to examine both morphological and physiological factors affecting the response of RGCs to high frequency electrical stimulation. Future steps will incorporate a large population of various RGCs subtypes as well as modeling primate retinal cells. We will investigate the impact of stimulation frequency and stimulus waveforms on synthetic retinal network response and axonal activation of RGCs. This positive correlation between the computationally-determined difference in firing rate of RGCs and the experimental results with patients is augmenting our fundamental knowledge of color perception, as well as enabling the opportunity to encode colors in the future generation of retinal prosthetics.

## Figures and Tables

**TABLE I:** Maximum Ionic Conductance Values for A2 and D1 Cells [S/CM ^2^ ]

	RGC types			
	A2		D1	
	Soma	Dendrite	Soma	Dendrite
g_Na_	0.35	0.1	0.2	0.08
g_K_	0.12	0.05	0.211	0.08
g_K,A_	3*g_K_	3*g_K_	3*g_K_	3*g_K_
g_K,Ca_	0.004*g_K_	0.004*g_K_	0.004*g_K_	0.004*g_K_
g_Ca_	0.137	0.05	0.013	0.01
g_h_	0	0	0.0001	3e–5
g_T_	0.004	0	0.0024	0.001

**TABLE II:** Maximum Ionic Conductance Values of the axon for A2 and D1 Cells [S/CM ^2^ ]

	AH	SOCB	NS	DA
g_Na_	0.8	2.4	0.9	0.8
g_K_	0.6	0.8	0.6	0.6
g_K,A_	3*g_K_	3*g_K_	3*g_K_	3*g_K_
